# Xeno-sensing activity of the aryl hydrocarbon receptor in human pluripotent stem cell-derived hepatocyte-like cells

**DOI:** 10.1038/srep21684

**Published:** 2016-02-22

**Authors:** Hye-Min Kim, Ji-Woo Kim, Youngjun Choi, Hang-Suk Chun, Ilkyun Im, Yong-Mahn Han, Chang-Woo Song, Seokjoo Yoon, Han-Jin Park

**Affiliations:** 1 Alternative Toxicological Methods Research Center, Korea Institute of Toxicology, Daejeon 34114, Republic of Korea; 2 Department of Biological Sciences, Korea Advanced Institute of Science and Technology, Daejeon 34141, Republic of Korea; 3 Jeonbuk Department of Inhalation Research, Korea Institute of Toxicology, Jeongeup, Jeollabuk-do, 56212, Republic of Korea; 4 System Toxicology Research Center, Korea Institute of Toxicology, Daejeon 34114, Republic of Korea; 5 Human and Environmental Toxicology, School of Engineering, University of Science and Technology, Daejeon 34113, Republic of Korea

## Abstract

Although hepatocyte-like cells derived from human pluripotent stem cells (hPSC-HLCs) are considered a promising model for predicting hepatotoxicity, their application has been restricted because of the low activity of drug metabolizing enzymes (DMEs). Here we found that the low expression of xenobiotic receptors (constitutive androstane receptor, CAR; and pregnane X receptor, PXR) contributes to the low activity of DMEs in hPSC-HLCs. Most CAR- and PXR-regulated DMEs and transporters were transcriptionally down-regulated in hPSC-HLC. Transcriptional expression of *CAR* and *PXR* was highly repressed in hPSC-HLCs, whereas mRNA levels of aryl hydrocarbon receptor (*AHR)* were comparable to those of adult liver. Furthermore, ligand-induced transcriptional activation was observed only at AHR in hPSC-HLCs. Bisulfite sequencing analysis demonstrated that promoter hypermethylation of *CAR* and *PXR* was associated with diminished transcriptional activity in hPSC-HLCs. Treatment with AHR-selective ligands increased the transcription of AHR-dependent target genes by direct AHR-DNA binding at the xenobiotic response element. In addition, an antagonist of AHR significantly inhibited AHR-dependent target gene expression. Thus, AHR may function intrinsically as a xenosensor as well as a ligand-dependent transcription factor in hPSC-HLCs. Our results indicate that hPSC-HLCs can be used to screen toxic substances related to AHR signaling and to identify potential AHR-targeted therapeutics.

The need for more predictive *in vitro* models of the human liver has been highlighted in toxicological studies. The typical hepatotoxicity assessments employing animal models do not adequately provide reliable data on the potential toxic effects of drugs and chemicals on humans as a result of interspecies differences in drug metabolism (or xenobiotic metabolism) and hepatotoxic responses^1^,^2^. While *in vitro* test systems employing human cellular models may overcome the problem of interspecies differences, the use of *in vitro* human cell line models is still limited. Freshly isolated or cryopreserved primary human hepatocytes (PHHs) are considered the ideal model for *in vitro* applications. However, the scarcity of fresh resources and tremendous variability in overall cell quality across different cell lots and donors constitute the main limitations^3^,^4^,^5^.

Recent advances in stem cell technology offer the opportunity to overcome the lack of *in vitro* models. *In vitro*-differentiated hepatocytes from human pluripotent stem cells (hPSCs) have emerged as the most attractive alternative source for hepatotoxicity prediction^6^,^7^. Several recent investigations show that human embryonic stem cell (hESC)- and human induced pluripotent stem cell (hiPSC)-derived hepatocyte-like cells (HLCs) exhibit predictive sensitivity for distinguishing hepatotoxicants from nontoxic compounds and the functional capacity to generate the relevant bioactive metabolites from known toxic agents^8^,^9^,^10^. The characteristics of hiPSC-HLCs also enable the prediction of inter-individual differences in drug metabolism and susceptibility on the basis of single nucleotide polymorphisms of cytochrome P450 (CYP) genes^11^. These findings of the predictive capacities of hPSC-HLCs wholly rely on the expression and functional activity of drug metabolizing enzymes (DMEs) and related drug transporters. To date, few studies have demonstrated that hPSC-HLCs are comparable to PHHs in terms of the expression and activity of DMEs and drug transporters^12^. However, the precise mechanism underlying how DMEs are induced and constitutively expressed in *in vitro*-differentiated HLCs remains poorly understood.

In the liver, expression and transcriptional regulation of DMEs and transporters is controlled by epigenetic factors (e.g., DNA methylation, histone modification, and miRNA) as well as by liver-enriched ligand-activated transcription factors^13^,^14^,^15^. A number of ligand-activated transcription factors, mostly nuclear receptors, exhibit miscellaneous xenobiotic binding capabilities and function as sensors of endogenous toxic metabolites and xenobiotics^16^,^17^. These receptors can induce the majority of genes involved in xenobiotic metabolism and transport by recognition of various ligands and are therefore also termed xenobiotic receptors. The major xenobiotic receptors, including constitutive androstane receptor (CAR, NR1I3), pregnane X receptor (PXR, NR1I2), and aryl hydrocarbon receptor (AHR), regulate the induction of a board spectrum of distinct and overlapping DMEs and transporters in the liver^18^,^19^,^20^. In particular, the induction profiles of a set of DMEs and transporters are strongly associated with the activation of these xenobiotic receptors. Moreover, each receptor exhibits species-specific differences in its target gene induction responses as well as in binding capability (and/or affinity) to its ligands^21^,^22^,^23^. As a result of these unique functions, various binding and activation assays employing CAR, PXR, and AHR are widely utilized as useful *in vitro* screening tools for predicting drug safety and xenobiotic-induced hepatotoxicity.

The purpose of this study was to determine the functional roles of CAR, PXR, and AHR as xenosensors and transcriptional regulators controlling xenobiotic metabolism in hPSC-HLCs. To this end, both hESCs and hiPSCs were differentiated into hepatocytes in which we assessed the mRNA expression and transcriptional activity of *CAR*, *PXR*, and *AHR*. We showed that a marked reduction in *CAR* and *PXR* transcript levels, as a result of promoter hypermethylation, led to diminished transcriptional activity in hPSC-HLCs. By contrast, the transcriptional activity of *AHR* was reproducible in hPSC-HLCs, which was tightly regulated by AHR-specific agonistic and antagonistic xenobiotics.

## Results

### *In vitro* differentiation of hESCs and hiPSC into HLCs

**Figure 1:**
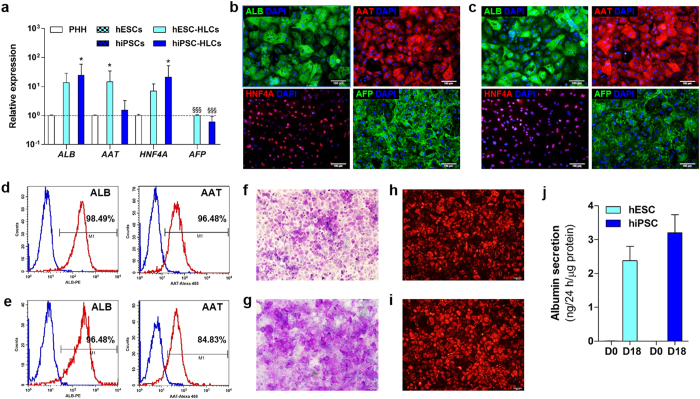
Characterization of hPSC-HLCs. All subsequent experiments were performed on both hESC-HLCs and hiPSC-HLCs at day 18, respectively. (**a**) Relative expression level of hepatic marker genes, *ALB*, *AAT*, *HNF4A*, and *AFP* in differentiated cells at day 0 and day 18 and PHHs were measured by qRT-PCR. Results represent mean ± SD (n = 3). ^*^p < 0.05, significant values in comparison with PHH; ^§§§^ p < 0.001, significant values in comparison with hESC-HLCs. (**b, c**) Immunofluorescence for ALB, AAT, HNF4A, and AFP in hESC-HLCs (**b**) and hiPSC-HLCs (**c**). Nuclei were counterstained with DAPI. Scale bar = 100 μm. (**d, e**) Flow cytometry analysis of ALB and AAT in hESC-HLCs (**d**) and hiPSC-HLCs (**e**). Blue line, isotype control; red line, target antibody. (**f, g**) Periodic acid-Schiff staining indicated hESC-HLCs (**f**) and hiPSC-HLCs (**g**) exhibiting cytoplasmic glycogen storage. Nuclei (light blue) were counterstained with hematoxylin. (**h, i**) Acetylated-low-density lipoprotein (Ac-LDL)-positive cells were detected in hESC-HLCs (**h**) and hiPSC-HLCs (**i**). (**j**) Albumin secretion by hPSC-HLCs was measured in the conditioned media by ELISA assay. Values represent means ± S.D (n = 3).

To investigate whether xenobiotic receptors are involved in the regulation of DME and transporters in hPSC-HLCs, we first differentiated hESCs and hiPSCs into HLCs in a stepwise manner. Then, hESC- and hiPSC-HLCs were phenotypically and functionally characterized. Transcriptional expression of hepatocyte marker genes including *AAT, ALB, AFP,* and *HNF4A* was prominently induced in hESC- and hiPSC-HLCs (Fig. 1a). Also, hESC- and hiPSC-HLCs were positive for AAT, ALB, AFP, and HNF4A (Fig. 1b,c). To determine hepatic differentiation efficiency, the proportion of ALB- and AAT- positive cells was measured by fluorescence-activated cell sorting (FACS). More than 96% of ALB-positive and at least 84% of AAT-positive cells were detected in both hPSC-HLC types (Fig. 1d,e). Furthermore, they exhibited various hepatic functions including glycogen synthesis, low density lipoprotein (LDL) uptake, and albumin secretion (Fig. 1f–j). Although minor differences were observed in marker gene expression and albumin secretion between hESC- and hiPSC-HLCs, the overall results indicate that both hESCs and hiPSCs were efficiently differentiated into hepatocyte-like cells.

### Down-regulation of genes regulated by xenobiotic receptors in hPSC-HLCs

**Figure 2:**
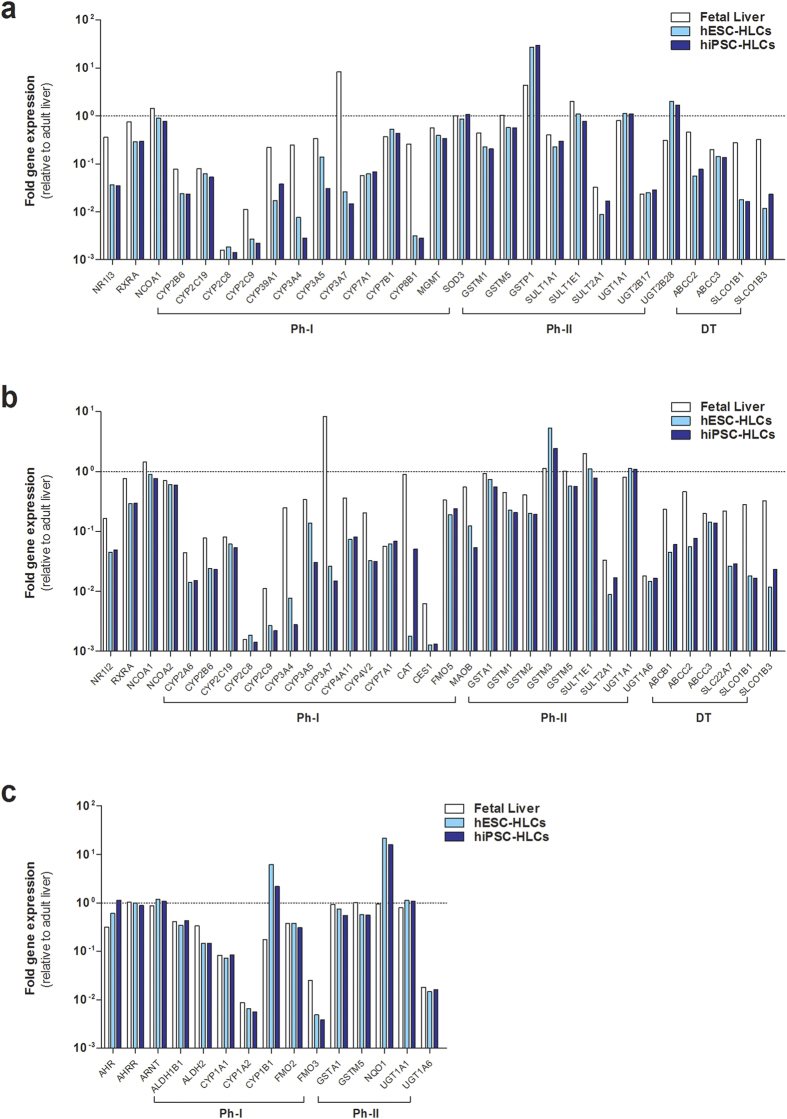
Quantitative gene expression analysis of genes regulated by CAR, PXR, and AHR. Quantitative comparison of CAR (**a**), PXR (**b**), and AHR (**c**) target genes in fetal liver, hESC-HLCs, and hiPSC-HLCs normalized to human adult liver (dotted line at value of 10^0^). The microarray data for adult and fetal liver are averaged across two independent sources, and for hESC-HLCs and hiPSC-HLCs are averaged across two different passages of origins, respectively. Gene expression levels in hPSC-HLCs ranged from 0.001- to 100-fold (**a, c**) and from 0.001- to 10-fold (**b**) as relative to adult liver levels. Ph-I, phase-I enzymes; Ph-II, phase-II enzymes; DT, drug transporters.

**Figure 3:**
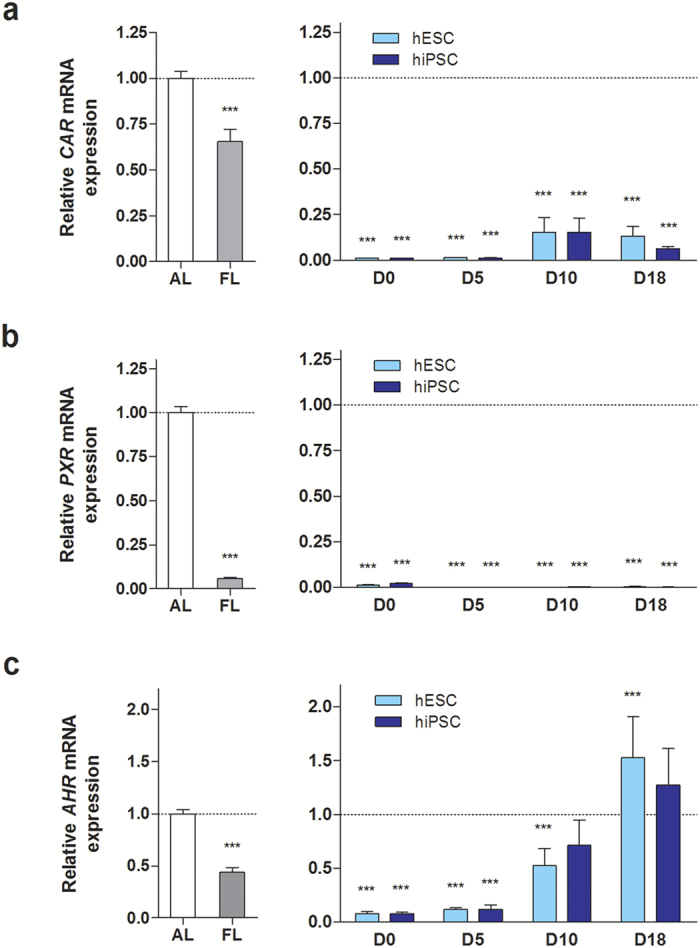
Changes in CAR, PXR, and AHR mRNA levels during hepatic differentiation of hESCs and hiPSCs. Expression levels of CAR (**a**), PXR (**b**), and AHR (**c**) mRNA in differentiated hESCs and hiPSCs at day 0, 5, 10, and 18 and in human adult and fetal liver were measured by qRT-PCR. Results represent mean ± SD (n = 3). ^**^p < 0.01, ^***^p < 0.001, significant values in comparison with adult liver. AL, adult liver; FL, fetal liver.

Next, we examined gene expression profiles for DMEs and transporters regulated by CAR, PXR, and AHR using microarray analysis. For the selection of target genes, we used Ingenuity pathway analysis to identify all genes directly and indirectly regulated by CAR, PXR, and AHR (Ingenuity^®^ System, www.ingenuity.com). The majority of CAR and/or PXR target genes belonging to phase-I enzymes and transporters in hESC- and hiPSC-HLCs were expressed at levels of less than 10% of adult liver (Fig. 2a). Transcript levels of most phase-II enzymes in hPSC-HLCs were comparable to those in fetal or adult hepatocytes, except glutathione S-transferase pi 1 (*GSTP1*) gene. Prominent expression of *GSTP1* and *AFP*, which are fetal hepatoblast markers, indicate that both HLCs differentiated from hESCs and hiPSCs still display fetal-like phenotypic characteristics (Figs 2a and 1a). The expression of *CAR* and *PXR* genes was also markedly reduced (<20-fold) in both hESC- and hiPSC-HLCs (Fig. 2a,b). These data imply that transcript level of *CAR* and *PXR* genes is closely related to the expression of phase-I enzyme and transporter genes in hPSC-HLCs. Unlike *CAR* and *PXR* mRNA levels, *AHR* mRNA levels in hESC- and hiPSC-HLCs were comparable to those of adult liver and were slightly higher than those of fetal liver, while the expression level of AHR-target genes varied from 0.0038-fold to 21-fold (Fig. 2c). To confirm the expression profiles of *CAR*, *PXR*, and *AHR* genes, mRNA levels of these receptors were measured during hepatic differentiation by qRT-PCR. *CAR* and *PXR* gene expression was significantly down-regulated in hESC- and hiPSC-HLCs (Fig. 3a,b). By contrast, *AHR* mRNA levels were markedly increased in hPSC-HLCs during hepatic differentiation and were higher on D18 of hepatic differentiation than in adult hepatocytes (Fig. 3c). These expression values of *CAR*, *PXR*, and *AHR* genes were consistent with the expression levels detected by microarray analysis (Fig. 2). Taken together, these results indicate that *CAR* and *PXR* genes and their target genes, including phase-I enzymes and transporters, are repressed in hESC- and hiPSC-HLCs.

### Epigenetic regulation of the expression of CAR, PXR, and AHR genes in hPSC-HLCs

**Figure 4:**
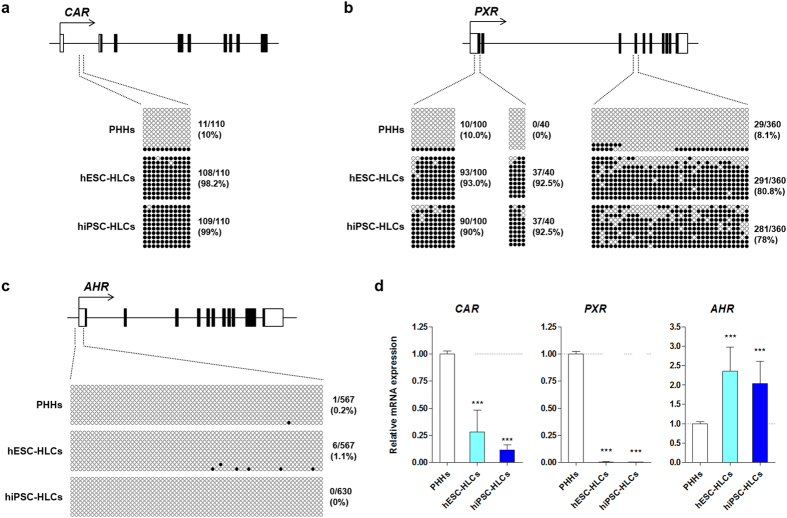
DNA methylation and CAR, PXR, and AHR mRNA expression in hPSC-HLCs and PHHs. DNA methylation status in the regulatory regions of *CAR* (**a**), *PXR* (**b**), and *AHR* (**c**) in hESC- and hiPSC-HLCs, and PHHs were analyzed by bisulfite sequencing. Each diagram represents the investigated locations containing CpG islands in promoter and gene body region. Each row represents the methylation status of each CpG in a series of 9 – 10 bacterial clones. The methylated and unmethylated CpG dinucleotides are represented as filled and open circles, respectively. (**d**) Expression levels of CAR, PXR and AHR mRNA in hESC- and hiPSC-HLCs and PHHs were measured by qRT-PCR. Results are the mean ± S.D (n = 3). ^***^p < 0.001, significant values in comparison with PHHs. PHHs, primary human hepatocytes.

To determine whether epigenetic modifications are associated with the regulation of *CAR*, *PXR*, and *AHR* genes in hPSC-HLCs, the DNA methylation states of CpG dinucleotides were investigated in the regulatory regions around the transcription start site (TSS) of these receptor genes. Bisulfide sequencing analysis revealed hypermethylation of CpG dinucleotides at the *CAR* and *PXR* promoter regions in hESC- and hiPSC-HLCs, but showed demethylation in PHHs (Fig. 4a,b). The DNA methylation frequency in the *CAR* promoter region, which includes 11 CpG sites, was 98.2%, 99%, and 10% in hESC-HLCs, hiPSC-HLCs, and PHHs, respectively (Fig. 4a). The *PXR* promoter region, containing two separated CpG rich regions, was hypermethylated (>90%) in both hESC- and hiPSC-HLCs but was hypomethylated (≤10%) in PHHs (Fig. 4b). A similar methylation pattern was also observed at the *PXR* exon 3 region (Fig. 4b). In hESCs, hypermethylation of CpG dinucleotides was also observed at *CAR* and *PXR* promoter regions (Supplementary Fig. 1a,b). In contrast to the *CAR* and *PXR* genes, the DNA methylation frequency at the *AHR* promoter region, which includes 63 CpG sites, was less than 2% in PHHs, hESC- and hiPSC-HLCs, and hESCs (Fig. 4c; Supplementary Fig. 1c). *AHR* transcripts were enriched in hESC- and hiPSC-HLCs relative to PHHs, but transcription of *CAR* and *PXR* was marginally activated in hESC- and hiPSC-HLCs (Fig. 4d). These results imply that the magnitude of DNA methylation closely correlates with the transcriptional expression of *CAR*, *PXR*, and *AHR* in hPSC-HLCs.

### Effects of treatment with respective CAR, PXR, and AHR ligands on target gene expression in hPSC-HLCs

**Figure 5:**
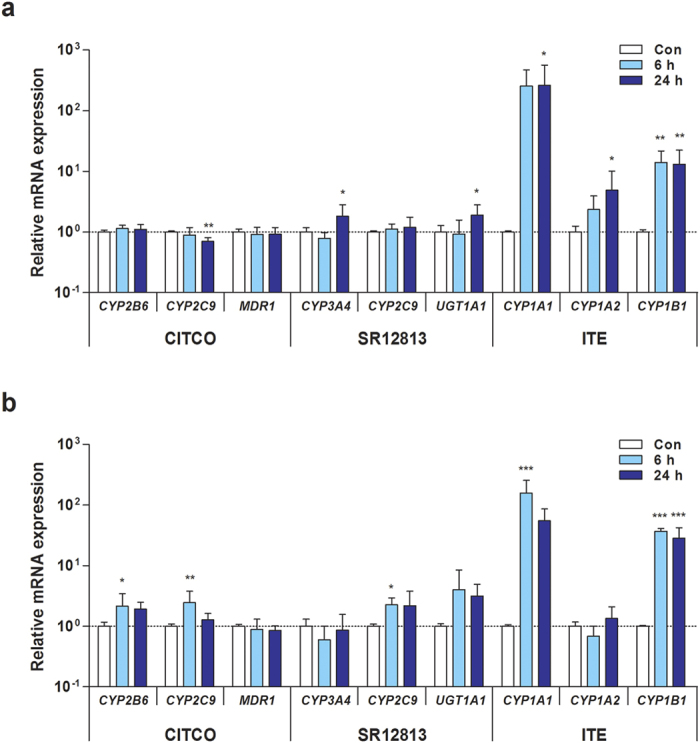
Ligand-induced transcriptional activation of CAR, PXR, and AHR in hPSC-HLCs. Inductions of CAR-, PXR-, and AHR-target genes in hESC-HLCs (**a**) and hiPSC-HLCs (**b**) treated with receptor-specific ligands for 6 h and 24 h were determined by qRT-PCR. CITCO (100 nM), SR12813 (200 nM), and ITE (500 nM) were used as agonists of CAR, PXR, and AHR, respectively. Results represent mean ± SD (n = 3). ^*^p < 0.05, ^**^p < 0.01, ^***^p < 0.001, significant values in comparison with 0.1% DMSO control (Con).

Next, to investigate whether activation of xenobiotic receptors influences the expression of downstream genes, hPSC-HLCs were cultured in the presence of respective ligands specific for CAR, PXR, and AHR. These receptors are ligand-activated transcription factors that regulate the expression of their specific target genes in a ligand-dependent manner^16^,^17^. Furthermore, 6-(4-chlorophenyl)imidazo[2,1-b][1,3]thiazole-5-carbaldehyde-O-(3,4-dichlorobenzyl)oxime (CITCO) and [[3,5-bis(1,1-dimethylethyl)-4-hydroxyphenyl]ethenylidene]bis-phosphonic acid tetraethyl ester (SR12813) are specific human CAR and PXR agonists, respectively^21^,^24^,^25^. As expected from the above results (Fig. 4), CITCO and SR12813 did not or marginally activate the transcription of CAR and PXR target genes, respectively, including *CYP2B6*, *CYP2C9*, *CYP3A4*, *MDR*, and *UGT1A1*, in hESC- and hiPSC-HLCs (Fig. 5a,b). CAR and PXR target gene expression levels were altered at levels less than 3-fold in both hPSC-HLC types after treatment with CITCO (100 nM) and SR12813 (200 nM) (Fig. 5a,b). By contrast, AHR-target genes, such as *CYP1A1* and *CYP1B1* except *CYP1A2*, were transcriptionally activated and robustly expressed in hESC- and hiPSC-HLCs in the presence of 2-(1H-Indol-3-ylcarbonyl)-4-thiazolecarboxylic acid methyl ester (ITE), which is an endogenous agonist for AHR (Fig. 5a,b). These results suggest that AHR is transcriptionally active in both hPSC-HLC types^26^.

### Occupancy of AHR on the promoter of target genes in hPSC-HLCs

**Figure 6:**
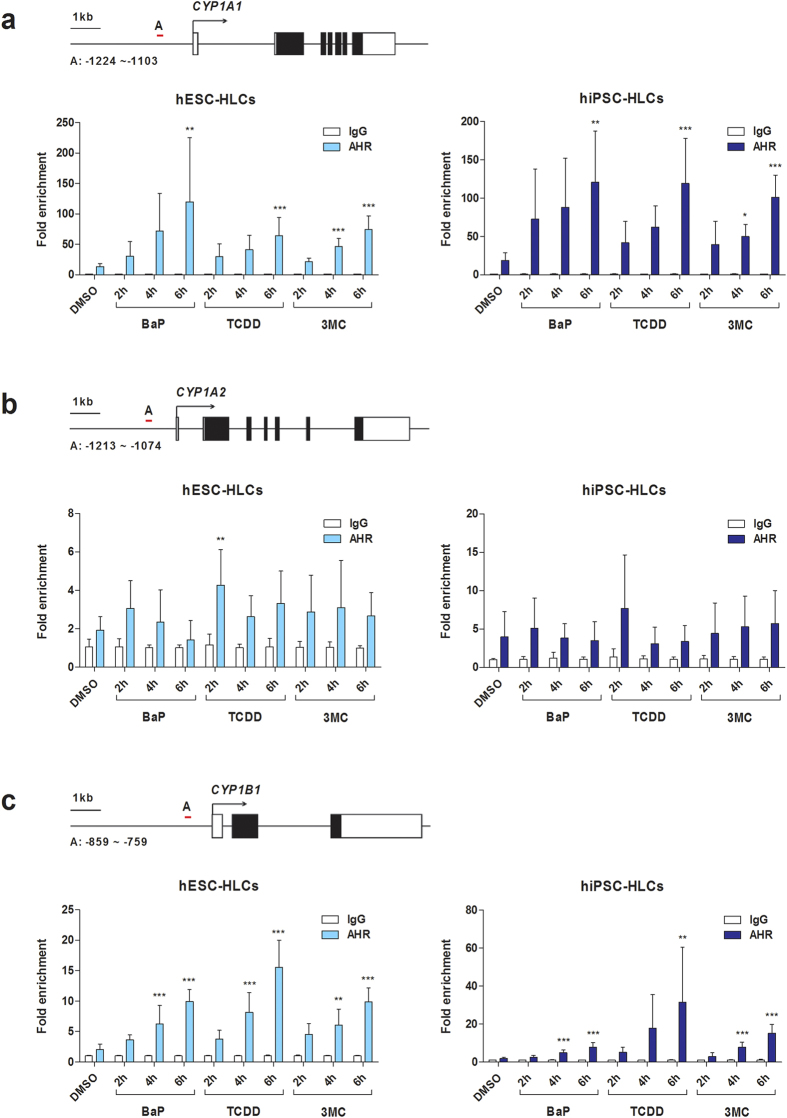
Ligand-dependent recruitment AHR to *CYP1A1* and *CYP1B1* promoter regions in hPSC-HLCs. hESC- and hiPSC-HLCs were treated with 1 μM BaP, 10 nM TCDD, 1 μM 3-MC, or DMSO (0.1%) for indicated amounts of time. ChIP assay were performed with primer pairs specific to the XRE-containing promoter regions of *CAP1A1* (**a**), *CYP1A2* (**b**), and *CYP1B1* (**c**) in hESC-HLCs (left panels) and hiPSC-HLCs (right panels) using real-time PCR. Primer pairs that amplify XRE-containing promoter regions as indicated in each diagram (upper panels). Numbers indicate nucleotide positions in relation to the transcription start site ( + 1, an arrow). Results are mean ± SD (n = 3) and are presented relative to isotype control (IgG = 1). ^*^p < 0.05, ^**^p < 0.01, ^***^p < 0.001, significant values in comparison with DMSO.

**Figure 7:**
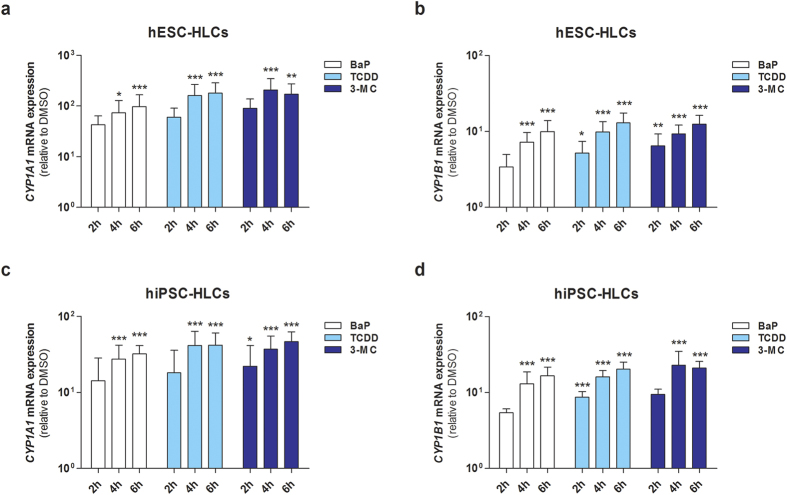
Ligand-dependent CYP1A1 and CYP1B1 induction in hPSC-HLCs. Cells were treated with 1 μM BaP, 10 nM TCDD, 1 μM 3-MC, or DMSO (0.1%) for indicated amounts of time. Ligand-induced CYP1A1 (**a, c**) and CYP1B1 (**b, d**) expression in hESC-HLCs (**a, b**) and hiPSC-HLCs (**c, d**) were determined by qRT-PCR analysis. Results are presented as the mean ± SD (n = 3). ^*^p < 0.05, ^**^p < 0.01, ^***^p < 0.001, significant values in comparison with DMSO.

AHR mediates myriad toxicological outcomes by sensing a variety of exogenous ligands such as benzo[a]pyrene (BaP), 2,3,7,8-tetrachlorodibenzodioxin (TCDD; also known as dioxin), and 3-methylcholanthrene (3-MC)^27^,^28^,^29^. Upon ligand binding, the activated AHR binds to a specific DNA recognition site known as the xenobiotic response element (XRE), thereby leading to target gene transcription^19^,^30^. To determine whether BaP, TCDD, and 3-MC mediate transcriptional regulation of *CYP1A* subfamily genes through an AHR-dependent mechanism in hPSC-HLCs, we assessed direct AHR-DNA binding to the XRE via a chromatin immunoprecipitation (ChIP) assay (Fig. 6). DNA fragments isolated from ChIP assays were analyzed by qRT-PCR, and GAPDH and immunoprecipitation with isotype IgG were used as negative controls. Treatment with BaP, TCDD, and 3-MC substantially increased AHR enrichment at the *CYP1A1* promoter region in a time-dependent manner in both hESC- and hiPSC-HLCs (Fig. 6a). AHR enrichment patterns at *CYP1B1* were similar to those observed at *CYP1A1* (Fig. 6c). By contrast, BaP, TCDD, and 3-MC had little effect on AHR recruitment to the *CYP1A2* promoter in hESC- and hiPSC-HLCs (Fig. 6b). To further determine whether AHR recruitment directly affects target gene transcription, we measured of *CYP1A1* and *CYP1B1* transcript levels in the presence of AHR ligands. In hESC- and hiPSC-HLCs, *CYP1A1* and *CYP1B1* transcripts levels were significantly increased after treatment with BaP, TCDD, and 3-MC (Fig. 7). These expression patterns were similar to the AHR enrichment patterns at *CYP1A1* and *CYP1B1* promoter regions (Fig. 6a,c). Thus, ligand-induced AHR recruitment results in AHR-mediated induction of *CYP1A1* and *CYP1B1* in hESC- and hiPSC-HLCs.

### Attenuation of AHR-dependent target gene expression by an AHR antagonist in hPSC-HLCs

**Figure 8:**
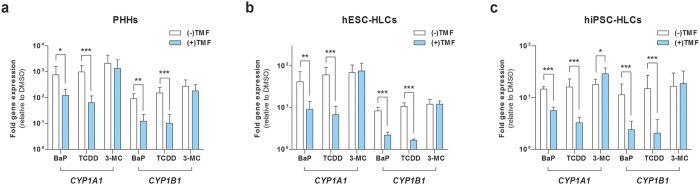
TMF antagonized AHR-mediated target gene expression. PHHs and hESC- and hiPSC-HLCs were treated with vehicle (DMSO), 1 μM BaP, 10 nM TCDD, and 1 μM 3-MC with or without 10 μM TMF for 6 h. Expression levels of AHR-responsive *CYP1A1* and *CYP1B1* in PHHs (**a**), hESC-HLCs (**b**), and hiPSC-HLCs (**c**) were determined by qRT-PCR analysis. Results are presented as the mean ± S.D (n = 3). *p < 0.05, ^**^p < 0.01, ^***^p < 0.001, significant values in comparison with cells treated with indicated compounds without TMF.

To determine whether the expression of *CYP1A1* and *CYP1B1* induced by toxic ligands is AHR-dependent or not, hPSC-HLCs were cultured with or without the AHR antagonist 6,2′,4′-trimethoxyflavone (TMF) for 6 h in the presence of BaP, TCDD, or 3-MC. In both hESC- and hiPSC-HLCs, TMF significantly inhibited BaP- and TCDD-induced *CYP1A1* and *CYP1B1* transcription, but did not significantly inhibit 3-MC-induced *CYP1A1* and *CYP1B1* transcription (Fig. 8b,c). No significant change was observed for TMF in 3-MC-treated hESC-HLCs (Fig. 8b). By contrast, TMF induced a significant, but slight, increase in *CYP1A1* transcription in 3-MC-treated hiPSC-HLCs (Fig. 8c). The inhibitory effect of TMF on AHR-dependent target gene induction was also examined in PHHs. TMF significantly reduced *CYP1A1* and *CYP1B1* transcript levels in BaP- and TCDD-treated PHHs (Fig. 8a), which was highly consistent with those observed in hPSC-HLCs (Fig. 8b,c). In 3-MC-treated PHHs, although TMF reduced *CYP1A1* and *CYP1B1* transcript levels, it was insignificant and less effective against 3-MC than against BaP or TCDD (Fig. 8a). The inhibitory effect of TMF against 3-MC-induced AHR-target gene transcription varied in hESC-HLCs, hiPSC-HLCs, and PHHs (Fig. 8). These results suggest that 3-MC induces *CYP1A1* and *CYP1B1* transcription via an AHR-independent mechanism. Taken together, our data indicate that the transcriptional activity of AHR was reproducible in both hESC- and hiPSC-HLCs, which was tightly regulated by AHR-specific agonists and antagonists.

## Discussion

Recent progress in technology and in the understanding of stem cell biology has enabled significant progress towards establishing alternative *in vitro* models based on hPSC-HLCs for drug safety and toxicology applications^6^,^31^,^32^. Several studies have demonstrated the applicability of hPSC-HLCs for predicting chemical toxicity, inter-individual drug responsiveness, and drug-induced liver injury^8^,^11^,^33^. Despite these advances, several major limitations associated with hPSC-HLCs, particularly incomplete drug metabolic activity, restrict their application in safety pharmacology and toxicology. Current hepatic differentiation processes yield functionally immature hPSC-HLCs displaying ‘fetal-like’ phenotypic characteristics and markedly reduced expression and activity of major DMEs and drug transporters^34^,^35^,^36^. DMEs and drug transporters enriched in hepatocytes play pivotal roles in the metabolism of endo- and xenobiotics as well as in the initiation of xenobiotic-induced hepatotoxicity. Xenobiotic-induced hepatotoxicity is widely accepted to be associated with the excessive formation of reactive xenobiotic metabolites catalyzed by phase-I enzymes, mostly CYP family enzymes, and the impairment of metabolite elimination mediated by phase-II enzymes and transporters^37^. Therefore, improvements in the expression and activity of hepatic enzymes and transporters involved in drug metabolism are required for the toxicological applications of hPSC-HLCs. However, the developmental pathways that regulate induction of DMEs and transporters are poorly investigated even though significant changes in DME expression are observed during liver ontogeny in various mammals^38^,^39^,^40^,^41^.

By contrast, xenobiotic-induced transcriptional regulation of DME and transporters in hepatocytes is relatively well elucidated. The induction of various DMEs and transporters is regulated by several ligand-activated transcription factors, namely, xenobiotic receptors, in response to promiscuous xenobiotics^16^,^20^,^37^. Despite its significance in xenobiotic metabolism as well as xenobiotic-induced hepatotoxicity, the function of xenobiotic receptors has not been investigated in detail in hPSC-HLCs. In this study, we investigated the function of three xenobiotic receptors, CAR, PXR, and AHR, in the regulation of DME and transporter expression in hPSC-HLCs. Gene expression analysis revealed a prominent reduction in the transcriptional level of the majority of phase-I enzymes and transporters regulated by CAR and PXR in both hESC- and hiPSC-HLCs (Fig. 2a,b). *CAR* mRNA levels were much lower in both hESC- and hiPSC-HLCs than in fetal liver as well as adult liver (Fig. 3a), and *PXR* mRNA was barely detectable (Fig. 3b). These results suggest that the considerably lower levels of DME and transporter expression may be due to the minimal expression of CAR and PXR. Indeed, neonatal activation of CAR results in long-term epigenetic memory and a permanent induction of CAR target genes in mouse liver^42^. Moreover, PXR activation by microbial byproducts promotes the metabolic maturation of hPSC-HLCs and fetal hepatocytes^43^.

The transcriptional regulatory mechanisms of CAR and PXR have been extensively studied; however, little is known about their expression. Epigenetic modification, for example, DNA methylation and histone modification, was recently shown to be involved in the regulation of the expression and activity of PXR^44^,^45^. A comparative analysis of epigenomes by Bonder *et al.* demonstrated that differential DNA methylation patterns in hepatic metabolism-related genes between human fetal and adult liver were tightly associated with developmental changes in their expression^46^. Thus, we examined the association between DNA methylation and the transcriptional activity of *CAR* and *PXR* in hPSC-HLCs. Bisulfite sequencing analysis revealed that promoter hypermethylation resulted in repression of *CAR* and *PXR* expression (Fig. 4a,b,d), which was likely associated with diminished transcriptional activity (Fig. 5). Both human CAR and PXR are capable of regulating the expression of various DMEs (e.g., CYP2B6, CYP2C9, CYP3A4, and UGT1A1) and drug transporters such as MDR1^47^. While PXR activation can nonselectively induce CYP2B6 and CYP3A4 expression, CAR preferentially induces CYP2B6 expression^48^. Notably, hESC- and hiPSC-HLCs treated with CITCO and SR12813, selective human CAR and PXR agonists^21^,^24^, showed only subtle changes in the expression levels of CAR and PXR target genes (Fig. 5a,b). The results indicated that CAR and PXR did not function as xenosensors or transcriptional regulators in both hESC- and hiPSC-HLCs. Taken together, our data suggest that the immature drug metabolism of hPSC-HLCs could be, at least in part, due to the lack of transcriptional activity of *CAR* and *PXR* as a result of promoter hypermethylation.

AHR is a member of the HLH (helix-loop-helix)-PER-ARNT-SIM (bHLH-PAS) protein family of heterodimeric transcription factors that function as sensors of extracellular signals and environmental stresses^19^,^49^. This xenobiotic receptor is capable of sustained hyperactivation via a number of polycyclic aromatic hydrocarbons (e.g., BaP), planar halogenated aromatic hydrocarbons (e.g., dioxins), and endogenous chemicals, which mediates diverse toxicological responses^27^. The importance of AHR activation in the critical stages of tumorigenesis is an intense focus of academic research^28^,^29^. Although extensive *in vivo* rodent studies have provided valuable insights into AHR-mediated toxicity, interspecies differences in AHR ligand binding affinity between humans and rodents have created a need for reliable human cell models^28^,^50^,^51^.

In the present study, we demonstrated that the intrinsic AHR function as a xenobiotic sensor, as well as a ligand-activated transcriptional factor, was reproducible in hPSC-HLCs. *AHR* mRNA expression levels in hESC- and hiPSC-HLCs were comparable to those of PHHs as well as adult liver (Figs 2c and 3c), and the DNA methylation pattern of the *AHR* promoter was similar to that in PHHs (Fig. 4c). Activation of AHR by an endogenous agonist and several toxic substances, including BaP, TCDD, and 3-MC, resulted in prominent induction of its target genes, including *CYP1A1* and *CYP1B1*, in hPSC-HLCs (Figs 5 and 7). Moreover, ChIP assays clearly demonstrated direct AHR-DNA binding at the XRE motif in the *CYP1A1* and *CYP1B1* promoters in response to BaP, TCDD, and 3-MC treatment (Fig. 6a,c). In contrast with *CYP1A1* and *CYP1B1*, constitutive *CYP1A2* mRNA expression was barely detectable in hESC- and hiPSC-HLCs (Fig. 2c). AHR agonists, including BaP, 3-MC, and TCDD, did not significantly induce AHR recruitment to the *CYP1A2* promoter (Fig. 6b). Our previous study demonstrated that the low expression of *CYP1A2* in hESC-HLCs was associated with the hypermethylation of CpG sites and histone modifications such as enrichment of repressive histone mark histone H3 trimethylated at lysine 27^52^. Therefore, it suggests that these epigenetic modulations are involved in the inhibition of AHR recruitment to the *CYP1A2* promoter in hPSC-HLCs.

Recent reports have highlighted the multiple physiological functions of AHR beyond that of a xenobiotic sensor and transcriptional regulator controlling xenobiotic metabolism. These newly identified functional roles of AHR in cancer, inflammation, and adaptive immunity^28^,^53^,^54^ suggest that AHR antagonism may be a potential novel therapeutic target for cancer and immune disease. We have shown that TMF effectively inhibited AHR-mediated transcription of endogenous target genes independent of the cell type, even in the presence of BaP and TCDD (Fig. 8). By contrast, TMF showed different inhibitory effects against 3-MC among PHHs, hESC-HLCs, and hiPSC-HLCs, which could be explained by 3-MC-induced *CYP1A1* and *CYP1B1* transcription via an AHR-independent mechanism. Recently, several studies indicated that 3-MC directly activates estrogen receptor α, which is capable of modulating AHR-mediated target gene transcription^55^,^56^,^57^. Taken together, our studies on the effect of AHR agonists and antagonists demonstrated that hPSC-HLCs were capable of reproducing the intrinsic functions of AHR. Furthermore, our data showed no significant differences between hESC- and hiPSC-HLCs in terms of xeno-sensing and transcriptional activity of AHR. Thus, the derivative HLCs from both hESCs and hiPSCs may be a useful alternative model for predicting AHR-mediated toxicity and for identifying potential AHR-targeted therapeutics.

## Materials and Methods

### Ethics statement

The using hESCs and hiPSCs in the present study was approved by the ethics committee of Korea Institute of Toxicology (approval number: 2013-001) and approval of this research was reported to Korea Centers for Disease Control and Prevention. The methods were carried out in accordance with the approved guidelines.

### Hepatic differentiation

The hESCs (CHA-hES15 cell line) and hiPSCs were maintained as previously described^58^. Furthermore, hESCs and hiPSCs were differentiated into hepatocytes as previously described with some modifications^59^. Briefly, hESCs and hiPSCs were cultured for 4 days on Matrigel (Corning, Tewksbury, MA, USA) in mTeSR1 (Stem Cell Technologies, Vancouver, Canada). Thereafter, hESCs were incubated in RPMI-1640 (Lonza, Baltimore, MD, USA) containing 0.5 mg/ml bovine serum albumin (BSA, Sigma-Aldrich, St. Louis, MO, USA), 1 × B27 (Invitrogen, Carlsbad, CA, USA), 50 ng/ml activin A (Peprotech, Rocky Hill, NJ, USA), and 0.5 mM sodium butyrate (Sigma-Aldrich) for 1 day, and then further cultured for 4 days in the same medium except that the concentration of sodium butyrate was reduced to 0.1 mM. After treatment with activin A, cells were cultured in RPMI-1640 containing 0.5 mg/ml BSA, 1 × B27, 10 ng/ml fibroblast growth factor 4 (FGF4, Peprotech), and 10 ng/ml hepatocyte growth factor (HGF, Peprotech) for 5 days. Hepatic maturation was induced by culturing cells in hepatocyte culture medium (HCM, Lonza) supplemented with 10 ng/ml FGF4, 10 ng/ml HGF, 10 ng/ml oncostatin M (Peprotech), and 0.1 μM dexamethasone (Sigma-Aldrich) for 7 days. The culture media was changed daily.

### Characterization of hESC- and hiPSC-HLCs

FACS, immunocytochemistry, and functional assays were performed as described in the Supplementary Materials and Methods.

### Human liver samples, primary hepatocyte culture, and chemical dosing

Total RNA obtained from human fetal and adult liver was purchased from Clontech Laboratories (Palo Alto, CA) and Agilent Technologies (Palo Alto, CA), respectively. Primary human hepatocytes (BD Gentest™ Cryo Human Hepatocytes, BD Biosciences, Donor No. HFC 476 and 261) were plated in collagen I coated culture plates (Corning) using a Gentest^TM^ High Viability CryoHepatocyte Recovery Kit (Corning) according to the manufacturer’s instructions. Experiments were performed 24 h later. Chemicals were purchased from Sigma-Aldrich, Cayman Chemicals (Ann Arbor, MI), or Tocris Bioscience (Ellisville, MO). For CAR, PXR, and AHR activation, hESC- and hiPSC-HLCs were treated in HCM (Lonza) with 100 nM CITCO, 200 nM SR12813, 500 nM ITE, or 0.1% vol/vol DMSO (as a control) dissolved in culture medium for 6 and 24 h. For the activation and inhibition of AHR, hESC- and hiPSC-HLCs were treated in HCM (Lonza) with 1 μM BaP, 10 nM TCDD, 1 μM 3-MC, or 0.1% vol/vol DMSO with or without 10 μM TMF dissolved in culture medium for 6 h.

### Gene expression profiling

Total RNA was isolated from cells using a PureLink RNA Mini Kit (Life Technologies) according to the manufacturer’s instruction. RNA integrity was determined with a 2100 Bioanalyzer (Agilent Technologies, Santa Clara, CA). A GeneChip Human Genome U133 plus 2.0 Array (Affymetrix, Santa Clara, CA) was used for the microarray analysis. All steps of cDNA synthesis, biotin-labeling, fragmentation, hybridization, staining, washing, and scanning were performed according to the manufacturer’s instructions. Raw intensity values on the microarrays were normalized with Robust Multi-Array Analysis (RMA) and annotated using GeneSpring GX software, version 13.0 (Agilent Technologies). The known and putative target genes of xenobiotic receptors were identified and selected using Ingenuity pathway analysis (Ingenuity^®^ System, www.ingenuity.com).

### Quantitative real-time PCR (qRT-PCR)

Total RNA was isolated from cells using a PureLink RNA Mini Kit (Life Technologies) and reverse-transcribed using SuperScript II Reverse Transcriptase (Invitrogen) according to the manufacturer’s protocol. Gene expression levels were measured by real-time RT-PCR using Power SYBR Green PCR Master Mix (Applied Biosystems, Foster City, CA, USA). Relative expression levels were analyzed using a StepOnePlus Real-Time PCR System (Applied Biosystems) according to the manufacturer’s instructions. Triplicate PCR reactions were performed for each sample. The primers used for gene expression analysis are listed in Supplementary Table 1. For comparative quantification, results from real-time PCR were expressed as the relative fold change compared to control cells, after normalization against glyceraldehyde-3-phosphate dehydrogenase (*GAPDH*). The ΔCt (SΔCt) values were calculated as the difference between the Ct values of *GAPDH* and the target. The ΔCt value of control cells was used as the control ΔCt (CΔCt) value. Relative gene expression levels were determined using the formula 2^−(SΔCt−CΔCt)^.

### Bisulfite sequencing

Genomic DNA was isolated from cells using a G-DEX IIc Genomic DNA Extraction Kit (iNtRON Biotechnology, Gyeonggi-do, Korea) according to the manufacturer’s protocol. Bisulfite conversion was performed using an EZ DNA Methylation-Gold Kit (ZYMO RESEARCH, Orange, CA, USA) according to the manufacturer’s protocol. Bisulfite-specific PCR reactions were performed on a GeneAmp PCR System 9700 (Applied Biosystems) using the following protocol: 95 °C for 15 min, 50 cycles of 95 °C for 20 s, 55 °C for 40 s, 72 °C for 30 s, and extension at 72 °C for 10 min. The primer sequences used for PCR are listed in Supplementary Table 2. PCR products were purified using a MEGAquick-spin Total Fragment DNA Purification Kit (iNtRON Biotechnology), cloned into pGEM T vector (Promega, Madison, WI, USA), and sequenced using an ABI 3730XL capillary DNA sequencer (Applied Biosystems). The methylation state of the CpG sites was determined from the sequence data by using QUMA (QUantification tool for Methylation Analysis) software^60^.

### Chromatin immunoprecipitation

ChIP experiments were performed using an EZ ChIP^TM^ chromatin immunoprecipitation kit (Millipore) according to the manufacturer’s protocol. Immunoprecipitation was performed with an AHR antibody (Thermo Fisher Scientific Pierce, PA5-29642). A rabbit IgG antibody (Abcam, ab37415) was used as an isotype control. The purified DNA was analyzed by real-time PCR. Quantitative PCR was performed on a StepOnePlus Real-Time PCR System (Applied Biosystems) according to the manufacturer’s instructions. The primer sequences used for PCR are listed in Supplementary Table 3. Triplicate PCR reactions were performed for each sample. ChIP-quantitative PCR results were calculated using the ΔΔCt method. The Ct value of the respective ChIP fraction was normalized against the Ct value of the input DNA fraction (ΔCt). Then, the Ct value of the ChIP fraction was normalized to the Ct value of the IgG control (ΔΔCt). The fold enrichment of immunoprecipitation was calculated using 2^−∆∆Ct^.

### Statistical analysis

Data obtained from three independent experiments were expressed as mean ± standard deviation (SD) and statistically analyzed by one-way analysis of variance (ANOVA) with Bonferroni’s multiple comparison test and t-test with Mann-Whitney test using GraphPad Prism software (San Diego, CA, USA). A *p*-value < 0.05 was considered significant.

## Additional Information

**How to cite this article**: Kim, H.-M. *et al.* Xeno-sensing activity of the aryl hydrocarbon receptor in human pluripotent stem cell-derived hepatocyte-like cells. *Sci. Rep.* **6**, 21684; doi: 10.1038/srep21684 (2016).

## Supplementary Material

Supplementary Information

## References

[b1] Martignoni M., Groothuis G. M. & de Kanter R. Species differences between mouse, rat, dog, monkey and human CYP-mediated drug metabolism, inhibition and induction. Expert Opin Drug Metab Toxicol. 2, 875–94 (2006). 17125407 10.1517/17425255.2.6.875

[b2] Cohen A. J. Critical review of the toxicology of coumarin with special reference to interspecies differences in metabolism and hepatotoxic response and their significance to man. Food Cosmet Toxicol. 17, 277–89 (1979). 387555 10.1016/0015-6264(79)90289-x

[b3] Gomez-Lechon M. J., Donato M. T., Castell J. V. & Jover R. Human hepatocytes in primary culture: the choice to investigate drug metabolism in man. Curr Drug Metab. 5, 443–62 (2004). 15544436 10.2174/1389200043335414

[b4] Hewitt N. J. *et al.* Primary hepatocytes: current understanding of the regulation of metabolic enzymes and transporter proteins, and pharmaceutical practice for the use of hepatocytes in metabolism, enzyme induction, transporter, clearance, and hepatotoxicity studies. Drug Metab Rev. 39, 159–234 (2007). 17364884 10.1080/03602530601093489

[b5] Guguen-Guillouzo C. & Guillouzo A. General review on *in vitro* hepatocyte models and their applications. Methods Mol Biol. 640, 1–40 (2010). 20645044 10.1007/978-1-60761-688-7_1

[b6] Szkolnicka D., Zhou W., Lucendo-Villarin B. & Hay D. C. Pluripotent stem cell-derived hepatocytes: potential and challenges in pharmacology. Annu Rev Pharmacol Toxicol. 53, 147–59 (2013). 23294308 10.1146/annurev-pharmtox-011112-140306

[b7] Mann D. A. Human induced pluripotent stem cell-derived hepatocytes for toxicology testing. Expert Opin Drug Metab Toxicol. 11, 1–5 (2015). 25385341 10.1517/17425255.2015.981523

[b8] Sirenko O., Hesley J., Rusyn I. & Cromwell E. F. High-Content Assays for Hepatotoxicity Using Induced Pluripotent Stem Cell–Derived Cells. Assay and drug development technologies. 12, 43–54 (2014). 24229356 10.1089/adt.2013.520 PMC3934660

[b9] Yildirimman R. *et al.* Human embryonic stem cell derived hepatocyte-like cells as a tool for *in vitro* hazard assessment of chemical carcinogenicity. Toxicol Sci. 124, 278–90 (2011). 21873647 10.1093/toxsci/kfr225 PMC3216410

[b10] Medine C. N. *et al.* Developing high-fidelity hepatotoxicity models from pluripotent stem cells. Stem Cells Transl Med. 2, 505–9 (2013). 23757504 10.5966/sctm.2012-0138 PMC3697818

[b11] Takayama K. *et al.* Prediction of interindividual differences in hepatic functions and drug sensitivity by using human iPS-derived hepatocytes. Proc Natl Acad Sci USA. 111, 16772–7 (2014). 25385620 10.1073/pnas.1413481111 PMC4250156

[b12] Berger D. R., Ware B. R., Davidson M. D., Allsup S. R. & Khetani S. R. Enhancing the functional maturity of induced pluripotent stem cell-derived human hepatocytes by controlled presentation of cell-cell interactions *in vitro*. Hepatology. 61, 1370–81 (2015). 25421237 10.1002/hep.27621

[b13] Ieiri I., Hirota T., Takane H. & Higuchi S. Epigenetic regulation of genes encoding drug-metabolizing enzymes and transporters; DNA methylation and other mechanisms. Current drug metabolism. 9, 34–38 (2008). 18220569 10.2174/138920008783331130

[b14] Glubb D. M. & Innocenti F. Mechanisms of genetic regulation in gene expression: examples from drug metabolizing enzymes and transporters. Wiley Interdiscip Rev Syst Biol Med. 3, 299–313 (2011). 20865777 10.1002/wsbm.125

[b15] Godoy P. *et al.* Recent advances in 2D and 3D *in vitro* systems using primary hepatocytes, alternative hepatocyte sources and non-parenchymal liver cells and their use in investigating mechanisms of hepatotoxicity, cell signaling and ADME. Arch Toxicol. 87, 1315–530 (2013). 23974980 10.1007/s00204-013-1078-5 PMC3753504

[b16] di Masi A., De Marinis E., Ascenzi P. & Marino M. Nuclear receptors CAR and PXR: Molecular, functional, and biomedical aspects. Mol Aspects Med. 30, 297–343 (2009). 19427329 10.1016/j.mam.2009.04.002

[b17] Tolson A. H. & Wang H. Regulation of drug-metabolizing enzymes by xenobiotic receptors: PXR and CAR. Adv Drug Deliv Rev. 62, 1238–49 (2010). 20727377 10.1016/j.addr.2010.08.006 PMC2991607

[b18] Pascussi J.-M. *et al.* The tangle of nuclear receptors that controls xenobiotic metabolism and transport: crosstalk and consequences. Annu. Rev. Pharmacol. Toxicol. 48, 1–32 (2008). 17608617 10.1146/annurev.pharmtox.47.120505.105349

[b19] McIntosh B. E., Hogenesch J. B. & Bradfield C. A. Mammalian Per-Arnt-Sim proteins in environmental adaptation. Annu Rev Physiol. 72, 625–45 (2010). 20148691 10.1146/annurev-physiol-021909-135922

[b20] Li H. & Wang H. Activation of xenobiotic receptors: driving into the nucleus. Expert Opin Drug Metab Toxicol. 6, 409–26 (2010). 20113149 10.1517/17425251003598886 PMC2842451

[b21] Watkins R. E. *et al.* The human nuclear xenobiotic receptor PXR: structural determinants of directed promiscuity. Science. 292, 2329–33 (2001). 11408620 10.1126/science.1060762

[b22] Wei P., Zhang J., Egan-Hafley M., Liang S. & Moore D. D. The nuclear receptor CAR mediates specific xenobiotic induction of drug metabolism. Nature. 407, 920–3 (2000). 11057673 10.1038/35038112

[b23] Connor K. T. & Aylward L. L. Human response to dioxin: aryl hydrocarbon receptor (AhR) molecular structure, function, and dose-response data for enzyme induction indicate an impaired human AhR. J Toxicol Environ Health B Crit Rev. 9, 147–71 (2006). 16613807 10.1080/15287390500196487

[b24] Maglich J. M. *et al.* Identification of a novel human constitutive androstane receptor (CAR) agonist and its use in the identification of CAR target genes. J Biol Chem. 278, 17277–83 (2003). 12611900 10.1074/jbc.M300138200

[b25] Moore L. B. *et al.* Orphan nuclear receptors constitutive androstane receptor and pregnane X receptor share xenobiotic and steroid ligands. J Biol Chem. 275, 15122–7 (2000). 10748001 10.1074/jbc.M001215200

[b26] Song J. *et al.* A ligand for the aryl hydrocarbon receptor isolated from lung. Proc Natl Acad Sci USA. 99, 14694–9 (2002). 12409613 10.1073/pnas.232562899 PMC137481

[b27] Hu W., Sorrentino C., Denison M. S., Kolaja K. & Fielden M. R. Induction of cyp1a1 is a nonspecific biomarker of aryl hydrocarbon receptor activation: results of large scale screening of pharmaceuticals and toxicants *in vivo* and *in vitro*. Mol Pharmacol. 71, 1475–86 (2007). 17327465 10.1124/mol.106.032748

[b28] Murray I. A., Patterson A. D. & Perdew G. H. Aryl hydrocarbon receptor ligands in cancer: friend and foe. Nat Rev Cancer. 14, 801–14 (2014). 25568920 10.1038/nrc3846 PMC4401080

[b29] Denison M. S., Soshilov A. A., He G., DeGroot D. E. & Zhao B. Exactly the same but different: promiscuity and diversity in the molecular mechanisms of action of the aryl hydrocarbon (dioxin) receptor. Toxicol Sci. 124, 1–22 (2011). 21908767 10.1093/toxsci/kfr218 PMC3196658

[b30] Bacsi S. G., Reisz-Porszasz S. & Hankinson O. Orientation of the heterodimeric aryl hydrocarbon (dioxin) receptor complex on its asymmetric DNA recognition sequence. Mol Pharmacol. 47, 432–8 (1995). 7700240

[b31] Kia R. *et al.* Stem cell-derived hepatocytes as a predictive model for drug-induced liver injury: are we there yet? Br J Clin Pharmacol. 75, 885–96 (2013). 22703588 10.1111/j.1365-2125.2012.04360.x PMC3612706

[b32] Greenhough S., Medine C. N. & Hay D. C. Pluripotent stem cell derived hepatocyte like cells and their potential in toxicity screening. Toxicology. 278, 250–255 (2010). 20674645 10.1016/j.tox.2010.07.012

[b33] Ware B. R., Berger D. R. & Khetani S. R. Prediction of Drug-Induced Liver Injury in Micropatterned Co-cultures Containing iPSC-Derived Human Hepatocytes. Toxicol Sci. 145, 252–62 (2015). 25716675 10.1093/toxsci/kfv048

[b34] Baxter M. *et al.* Phenotypic and functional analyses show stem cell-derived hepatocyte-like cells better mimic fetal rather than adult hepatocytes. J Hepatol. 62, 581–9 (2015). 25457200 10.1016/j.jhep.2014.10.016 PMC4334496

[b35] Wobus A. M. & Loser P. Present state and future perspectives of using pluripotent stem cells in toxicology research. Arch Toxicol. 85, 79–117 (2011). 21225242 10.1007/s00204-010-0641-6 PMC3026927

[b36] Schwartz R. E., Fleming H. E., Khetani S. R. & Bhatia S. N. Pluripotent stem cell-derived hepatocyte-like cells. Biotechnol Adv. 32, 504–13 (2014). 24440487 10.1016/j.biotechadv.2014.01.003 PMC4043206

[b37] Omiecinski C. J., Vanden Heuvel J. P., Perdew G. H. & Peters J. M. Xenobiotic metabolism, disposition, and regulation by receptors: from biochemical phenomenon to predictors of major toxicities. Toxicol Sci. 120 **Suppl 1**, S49–75 (2011). 21059794 10.1093/toxsci/kfq338 PMC3145385

[b38] Hart S. N., Cui Y., Klaassen C. D. & Zhong X. B. Three patterns of cytochrome P450 gene expression during liver maturation in mice. Drug Metab Dispos. 37, 116–21 (2009). 18845660 10.1124/dmd.108.023812 PMC2683655

[b39] Lee J. S. *et al.* Transcriptional ontogeny of the developing liver. BMC Genomics. 13, 33 (2012). 22260730 10.1186/1471-2164-13-33 PMC3306746

[b40] Ise R. *et al.* Expression of cytochromes p450 in fetal, infant, and juvenile liver of cynomolgus macaques. Drug Metab Pharmacokinet. 26, 621–6 (2011). 21844657 10.2133/dmpk.DMPK-11-NT-057

[b41] Hines R. N. Ontogeny of human hepatic cytochromes P450. J Biochem Mol Toxicol. 21, 169–75 (2007). 17936930 10.1002/jbt.20179

[b42] Chen W. D. *et al.* Neonatal activation of the nuclear receptor CAR results in epigenetic memory and permanent change of drug metabolism in mouse liver. Hepatology. 56, 1499–509 (2012). 22488010 10.1002/hep.25766 PMC3407349

[b43] Avior Y. *et al.* Microbial-derived lithocholic acid and vitamin K2 drive the metabolic maturation of pluripotent stem cells-derived and fetal hepatocytes. Hepatology. 62, 265–78 (2015). 25808545 10.1002/hep.27803

[b44] Tian Y. Epigenetic regulation of pregnane X receptor activity. Drug Metab Rev. 45, 166–72 (2013). 23600685 10.3109/03602532.2012.756012

[b45] Habano W. *et al.* Involvement of promoter methylation in the regulation of Pregnane X receptor in colon cancer cells. BMC Cancer. 11, 81 (2011). 21342487 10.1186/1471-2407-11-81 PMC3053268

[b46] Bonder M. J. *et al.* Genetic and epigenetic regulation of gene expression in fetal and adult human livers. BMC Genomics. 15, 860 (2014). 25282492 10.1186/1471-2164-15-860 PMC4287518

[b47] Wang Y. M., Ong S. S., Chai S. C. & Chen T. Role of CAR and PXR in xenobiotic sensing and metabolism. Expert Opin Drug Metab Toxicol. 8, 803–17 (2012). 22554043 10.1517/17425255.2012.685237 PMC3593589

[b48] Faucette S. R. *et al.* Differential regulation of hepatic CYP2B6 and CYP3A4 genes by constitutive androstane receptor but not pregnane X receptor. J Pharmacol Exp Ther. 317, 1200–9 (2006). 16513849 10.1124/jpet.105.098160

[b49] Bersten D. C., Sullivan A. E., Peet D. J. & Whitelaw M. L. bHLH-PAS proteins in cancer. Nat Rev Cancer. 13, 827–41 (2013). 24263188 10.1038/nrc3621

[b50] Boffetta P., Mundt K. A., Adami H. O., Cole P. & Mandel J. S. TCDD and cancer: a critical review of epidemiologic studies. Crit Rev Toxicol. 41, 622–36 (2011). 21718216 10.3109/10408444.2011.560141 PMC3154583

[b51] Cole P., Trichopoulos D., Pastides H., Starr T. & Mandel J. S. Dioxin and cancer: a critical review. Regul Toxicol Pharmacol. 38, 378–88 (2003). 14623487 10.1016/j.yrtph.2003.08.002

[b52] Park H. J. *et al.* Differences in the Epigenetic Regulation of Cytochrome P450 Genes between Human Embryonic Stem Cell-Derived Hepatocytes and Primary Hepatocytes. PLoS One. 10, e0132992 (2015). 26177506 10.1371/journal.pone.0132992 PMC4503736

[b53] Quintana F. J. & Sherr D. H. Aryl hydrocarbon receptor control of adaptive immunity. Pharmacol Rev. 65, 1148–61 (2013). 23908379 10.1124/pr.113.007823 PMC3799235

[b54] Vondracek J., Umannova L. & Machala M. Interactions of the aryl hydrocarbon receptor with inflammatory mediators: beyond CYP1A regulation. Curr Drug Metab. 12, 89–103 (2011). 21401513 10.2174/138920011795016827

[b55] Wihlen B., Ahmed S., Inzunza J. & Matthews J. Estrogen receptor subtype- and promoter-specific modulation of aryl hydrocarbon receptor-dependent transcription. Mol Cancer Res. 7, 977–86 (2009). 19470599 10.1158/1541-7786.MCR-08-0396

[b56] Abdelrahim M. *et al.* 3-Methylcholanthrene and other aryl hydrocarbon receptor agonists directly activate estrogen receptor alpha. Cancer Res. 66, 2459–67 (2006). 16489053 10.1158/0008-5472.CAN-05-3132

[b57] Safe S. 3-methylcholanthrene induces differential recruitment of aryl hydrocarbon receptor to human promoters. Toxicol Sci. 117, 1–3 (2010). 20651249 10.1093/toxsci/kfq193 PMC2923292

[b58] Lee J. E. *et al.* Evaluation of 28 human embryonic stem cell lines for use as unrelated donors in stem cell therapy: implications of HLA and ABO genotypes. Cell Transplant. 19, 1383–95 (2010). 20587141 10.3727/096368910X513991

[b59] Liu H., Ye Z., Kim Y., Sharkis S. & Jang Y. Y. Generation of endoderm-derived human induced pluripotent stem cells from primary hepatocytes. Hepatology. 51, 1810–9 (2010). 20432258 10.1002/hep.23626 PMC2925460

[b60] Kumaki Y., Oda M. & Okano M. QUMA: quantification tool for methylation analysis. Nucleic Acids Res. 36, W170–5 (2008). 18487274 10.1093/nar/gkn294 PMC2447804

