# Supplementary material for: Xeno-sensing activity of the aryl hydrocarbon receptor in human pluripotent stem cell-derived hepatocyte-like cells
Source: Sci Rep. 2016 Feb 22;6:21684. doi: 10.1038/srep21684 (PMC4761945; doi:10.1038/srep21684)
Supplement: Supplementary Information [file srep21684-s1.pdf]

Xeno-sensing activity of the aryl hydrocarbon receptor in human pluripotent stem cell-derived hepatocyte-like cells

Hye-Min Kim[1], Ji-Woo Kim[1], Youngjun Choi[1, 5], Hang-Suk Chun[1], Ilkyun Im[2], Yong-Mahn Han[2], Chang-Woo Song[3, 5], Seokjoo Yoon[4, 5, +], & Han-Jin Park[1, *, +]

[1]Alternative Toxicological Methods Research Center, Korea Institute of Toxicology, Daejeon 34114, Republic of Korea

[2]Department of Biological Sciences, Korea Advanced Institute of Science and Technology, Daejeon 34141, Republic of Korea

[3]Jeonbuk Department of Inhalation Research, Korea Institute of Toxicology, Jeongeup, Jeollabuk-do, 56212, Republic of Korea

[4]System Toxicology Research Center, Korea Institute of Toxicology, Daejeon 34114, Republic of Korea

[5]Human and Environmental Toxicology, School of Engineering, University of Science and Technology, Daejeon 34113, Republic of Korea

[*]corresponding. sjyoon@kitox.re.kr or hjpark@kitox.re.kr

[+]These authors contributed equally to this work

Supplementary Information

Supplementary Materials and Methods

Fluorescence-activated cell sorting (FACS)

Cells were dissociated by incubation with 0.05% collagenase IV (Invitrogen) at 37℃ for 15 min followed by incubation with Accutase (Innovative Cell Technologies, San Diego, CA, USA) at 37℃ for 15 min. Dissociated cells were fixed and permeabilized with Foxp3 Fixation/Permeabilization solution (eBioscience, San Diego, CA, USA) for 1 h at room temperature (RT). One microgram each of mouse anti-ALB (R&D Systems) and rabbit anti-AAT (Abcam, Cambridge, UK) was conjugated using a Zenon R-Phycoerythrin Mouse IgG2a Labeling Kit and Zenon Alexa Fluor 488 Rabbit IgG Labeling Kit (Invitrogen), respectively, according to the manufacturer's instructions. Cells were incubated at RT for 1 h with each labeled antibody. Cells were also labeled with the isotype control as a negative control. Flow cytometry was performed using BD FACS Calibur system (BD Biosciences).

Immunocytochemistry

Cells were fixed in 4% formaldehyde (Sigma-Aldrich) for 30 min at RT, rinsed three times in PBS containing 0.1% Tween 20 (PBST) for 10 min, permeabilized in PBS containing 0.5% saponin (Sigma-Aldrich; ALB, AAT, and CYP1A1) or 0.1% Triton X-100 (Sigma-Aldrich; HNF4A) for 15 min, and blocked for 1 h in PBS containing 4% normal goat serum (Jackson ImmunoResearch, West Grove, PA, USA). Cells were incubated overnight at 4℃ with the following primary antibodies diluted in PBS containing 4% normal goat serum: rabbit anti-ALB (1:50; Dako, Glostrup, Denmark), rabbit anti-AAT (1:200; Abcam), mouse anti-HNF4A (1:200; Abcam), rabbit anti-CYP1A1 (1:200; Abcam). Cells were rinsed six times in PBST for 10 min each. Thereafter, cells were incubated for 1 h at RT with appropriate secondary antibodies diluted in PBST as follows: Alexa Fluor 488 goat anti-rabbit IgG and Alexa Fluor 488 or 594 goat anti-mouse IgG (1:200; Invitrogen). Cells were washed six times in PBST and stained with 4'-6-diamidino-2-phenylindole (DAPI, Sigma-Aldrich).

Periodic acid-Schiff (PAS) staining

Cells were fixed in 4% formaldehyde for 30 min, rinsed three times in PBST for 10 min, permeabilized with PBS containing 0.1% Triton X-100 for 15 min, and rinsed three times in PBST. Samples were stained using a PAS staining system (Sigma-Aldrich) according to the manufacturer's instructions and observed under white light using an inverted microscope.

Acetylated-low density lipoprotein (Ac-LDL) uptake

Cells were incubated with 10 μg/ml 1,1'-dioctadecyl-3,3,3',3'-tetramethylindocarbocyanine-labeled Ac-LDL (Life Technologies, Carlsbad, CA, USA) for 5 h. Red fluorescence was visualized by fluorescence microscopy.

Enzyme-linked immunosorbent assay (ELISA)

Culture supernatants were collected 24 h after fresh medium was added and the amount of secreted albumin was measured using a Human Albumin ELISA Quantitation Kit (Bethyl Laboratories, Montgomery, TX, USA) according to the manufacturer's instructions. The mean amount of secreted albumin was measured using 100 µl of culture supernatant from two culture dishes and calculated according to each standard followed by normalization to the protein content. Protein concentrations were determined using a Bio-Rad Protein Assay kit (Bio-Rad).

Supplementary figure legends

Supplementary Figure S1. **DNA methylation in the regulatory region of *CAR*, *PXR*, and *AHR* genes in hESCs**. DNA methylation status in the regulatory regions of *CAR* (**a**), *PXR* (**b**), and *AHR* (**c**) in hESCs was analyzed by bisulfite sequencing. Each diagram represents the investigated locations containing CpG islands in promoter and gene body region. Each row represents the methylation status of each CpG in a series of 9 – 10 bacterial clones. The methylated and unmethylated CpG dinucleotides are represented as filled and open circles, respectively.

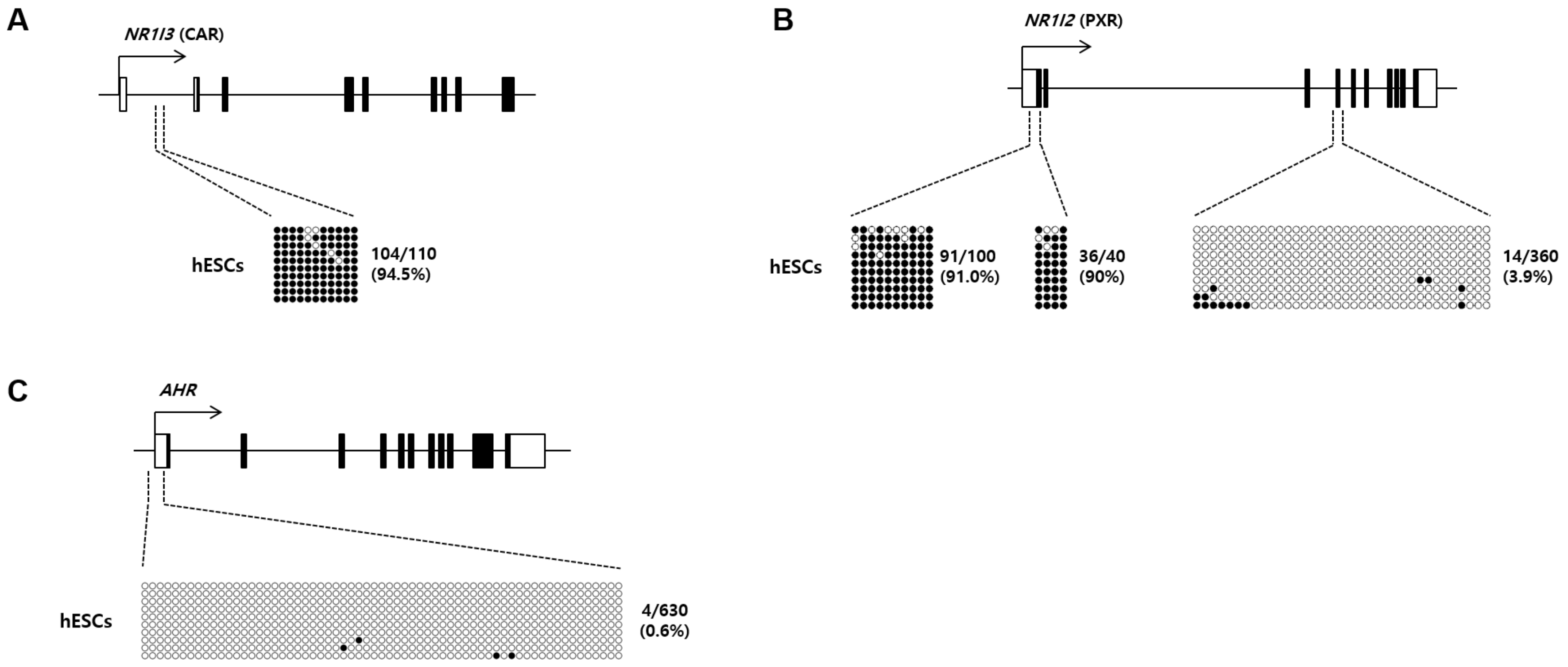

Supplementary Figure S1. Kim et al.

Supplementary Tables

Supplementary Table S1. Primers used for gene expression analyses

Gene	NCBI No.	Forward primer	Reverse primer	Product size (bp)
ALB	NM_000477	gagaccagaggttgatgtgatg	agttccggggcataaaagtaag	114
AAT	NM_000295	gaagtcaaggacaccgagga	gctggcagaccttctgtctt	261
HNF4A	NM_000457	cgagcagatccagttcatca	tcacacatctgtccgttgct	201
AFP	NM_001134	agcttggtggtggatgaa	tctgcaatgacagcctcaag	182
AHR	NM_001621.4	caaatccttccaagcggcata	cgctgagcctaagaactgaaag	123
NR1I3	NM_001077470.1	ctgtcggcagaagccctggc	gcccccaggagtgtccggat	116
NR1I2	NM_003889.3	tgcgagatcacccggaagac	atgggagaaggtagtgtcaaagg	255
CYP1A1	NM_000499.3	aggcttttacatccccaagg	ttgtcgatagcaccatcagg	132
CYP1A2	NM_000761.3	atgggcaagcgccggtgtat	cagttgatggagaagcgcagcc	194
CYP1B1	NM_000104.3	acgtaccggccactatcact	ctccccacgacctgatcca	142
CYP2B6	NM_000767.4	ccggggatatggtgtgatctt	ccgaagtccctcatagtggtc	84
CYP2C9	NM_000771.3	tgaagaagagcagatggcctg	agatgacaggtgagaaaaggca	110
CYP3A4	NM_017460.5	gtggggcttttatgatggtca	gcctcagatttctcaccaacaca	272
UGT1A1	NM_000463.2	ggaatcaactgccttcaccaa	acaggactgtctgagggattttg	175
MDR1	NM_000927.4	gggagcttaacacccgactta	gccaaaatcacaagggttagctt	154
GAPDH	NM_002046	catgagaagtatgacaacagcct	agtccttccacgataccaaagt	113

Supplementary Table S2. Primers used for bisulfite sequencing

Gene	Primer sequence	Genomic region (TSS = +1)	No. of CpGs	Product size (bp)
AHR	F: gaagtattttggatttaggaagttt<br>R: ctactactattcataatacccaacc	+139 to +627	63	489
NR1I3 (CAR)	F: agatggggttttattatattggttag<br>R: atcacttaaaatcaaaaatttattttt	+858 to +1254	11	397
NR1I2 (PXR)	F: ggagaggaaagagtttttagttggt<br>R: taaatcccaaatccaacacttaaac	+1007 to +1493	4	487
	F: ttaagtgttggatttgggatttag<br>R: aaaataaaaaaacaacaacaaaacca	+1471 to +1969	10	499
	F: agttgagttttggatttttagggat<br>R: tcccccaaataactacctattacac	+29386 to +29877	36	492

F, forward; R, reverse; TSS, transcription start site

Supplementary Table S3. Primers used for chromatin immunoprecipitation

Gene	Primer sequence	Genomic region (TSS = +1)	Product size (bp)
CYP1A1	F: agctaggccatgccaaat<br>R: aagggtctaggtctgcgtgt	-1224 to -1103	121
CYP1B1	F: cccctcaagcaaggataaac<br>R: tgcagtgagccaagatcg	-1213 to -1074	100
CYP1A2	F: atatgactggagccgactttcc<br>R: ggcgaactttatcgggttga	-859 to -759	140

F, forward; R, reverse; TSS, transcription start site
